# Oscillatory Transcranial Electrical Stimulation and the Amplitude‐Modulated Frequency Dictate the Quantitative Features of Phosphenes

**DOI:** 10.1111/ejn.16658

**Published:** 2025-01-08

**Authors:** Che‐Yi Hsu, Tzu‐Ling Liu, Chi‐Hung Juan

**Affiliations:** ^1^ Institute of Cognitive Neuroscience National Central University Taiwan; ^2^ Cognitive Intelligence and Precision Healthcare Research Center National Central University Taiwan

**Keywords:** amplitude modulation (AM), oscillating transcranial direct current stimulation (otDCS), phosphene, transcranial alternating current stimulation (tACS)

## Abstract

Previous research demonstrated that transcranial alternating current stimulation (tACS) can induce phosphene perception. However, tACS involves rhythmic changes in the electric field and alternating polarity (excitatory vs. inhibitory phases), leaving the precise mechanism behind phosphene perception unclear. To disentangle the effects of rhythmic changes from those of alternating polarity, this study employs oscillatory transcranial direct current stimulation (otDCS), in which the current oscillation remains confined to either a positive or negative polarity, thereby eliminating the influence of polarity switching. We applied scalp electrical stimulations using both polarity‐switching (tACS) and non‐polarity‐switching (otDCS) methods, with anodal or cathodal polarities, targeting the occipital lobe. All stimulations were performed using sinusoidal or amplitude modulation (AM) waveforms at threshold or suprathreshold intensities. Our results show that tACS results in faster response times compared to cathodal otDCS, but not anodal otDCS, while anodal otDCS elicits greater brightness perception than both cathodal otDCS and tACS. Additionally, AM frequency induced a higher threshold than the sinusoidal frequency, and response times were slower in the AM condition across all positive, negative, and polarity‐switching stimulations. However, stimulation intensity in the anodal AM condition could influence speed ratings, unlike in cathodal or tACS conditions. Our findings reveal that both tACS and otDCS induce phosphenes, with significant differences between polarities and current oscillation types, indicating that both mechanisms are critical in phosphene induction. This study provides evidence linking phosphene occurrence to oscillatory current activity and highlights the robustness and impact of AM coding in visual perception.

## Graphical abstract

The current study reveals that the oscillatory nature of electrical stimulation, independent of alternating or direct current, is essential for inducing phosphenes. The results highlight that the underlying mechanism for electrically induced phosphenes may involve ephaptic neural synchronization. Moreover, the amplitude‐modulated waveform can override the carrier frequency in dictating participants' phosphene reports.

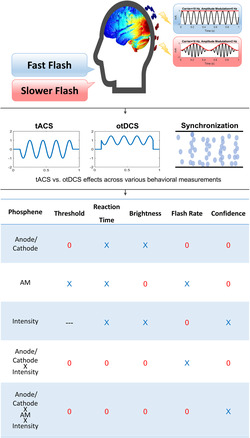

**Abbreviations**
AM: amplitude modulation
GTEN: Geodesic Transcranial Electrical Neuromodulation
MOBS: modified binary search
otDCS: oscillatory transcranial direct current stimulation
RT: response time
tACS: transcranial alternating current stimulation
tDCS: transcranial direct current stimulation
TES: transcranial electrical stimulation

## 1 Introduction

Aligning neuronal activity with the content of perception is a fundamental challenge in consciousness studies. This alignment helps address the “what‐it‐is‐like” problem (Nagel 1974) and lays the technical groundwork for developing virtual perception technologies, such as bionic eyes and auditory prostheses. Although the brain does not receive direct light stimulation, understanding the “visual code” that links brain activity with perception remains essential. One approach to investigating this connection is through phosphenes—the experience of perceiving light without external visual stimuli.

Research has shown that phosphene experiences can be induced through direct electrical stimulation of the visual cortex (Brindley and Lewin 1968; Foerster 1929; Fox et al. 2020) or by applying magnetic pulses over the visual cortex (Barker, Jalinous, and Freeston 1985; Meyer and Allen 1991). Notably, transcranial alternating current stimulation (tACS), which involves a current with alternating polarity, has also been demonstrated to elicit phosphenes (Kanai et al. 2008). Phosphenes induced by tACS are often perceived as repetitive flickering in the visual field. Previous studies have shown that the perception of tACS‐induced phosphenes may depend on the stimulation frequency (Evans et al. 2019; Evans, Palmisano, and Croft 2021; Hsu et al. 2023; Kanai et al. 2008; Turi et al. 2013).

In contrast to conventional tACS, amplitude‐modulated tACS (AM‐tACS) has recently been adopted for research, partly due to efforts to reduce phosphene occurrence during stimulation while exploring the effects of tACS on visual cognitive performance (Thiele et al. 2021). One strategy to achieve this involves embedding the target frequency within a rapidly oscillating tACS in amplitude modulation (AM) form (Thiele et al. 2021). This approach aims to prevent phosphene occurrence (Thiele et al. 2021) and minimize interference with surrounding neural activity during neural entrainment (Witkowski et al. 2016). Recent studies have highlighted the critical role of AM information in sensory encoding (Clarke, Longtin, and Maler 2015; Juan et al. 2021; Nguyen et al. 2019; Ryu et al. 2017). Notably, Hsu et al. (2023) provided direct insights into the effects of AM‐tACS on visual awareness, revealing the role of AM in tACS‐induced phosphene perception. This study applied AM‐tACS with carrier frequencies of 10, 14, 18 and 22 Hz, along with AM frequencies of 0, 2, and 4 Hz. The findings indicated that AM‐tACS‐induced phosphene perception exhibited higher thresholds and a slower flash rate, suggesting that AM frequency impacts phosphene perception more than carrier frequency. However, our understanding of how AM frequency influences visual perception remains in its early stages (Juan et al. 2021; Nguyen et al. 2019). In the present study, we build upon our previous work to investigate the neural mechanisms underlying phosphene perception induced by transcranial electrical brain stimulation.

To understand phosphene induction using tACS, note that tACS involves applying an alternating current. The polarities are designated as the “source” and “sink”, rhythmically switching between the two electrodes. Unlike phosphene induction through transcranial magnetic stimulation, where magnetic pulses trigger neural firing in the visual cortex, scalp electrical stimulation via tACS is not strong enough to directly elicit action potentials in the cortex (Asamoah, Khatoun, and Mc Laughlin 2019; Huang 2017; Vöröslakos et al. 2018). However, the oscillations of the electric field can modulate the rhythms of spontaneous neural firing, leading to synchronous excitation and inhibition during specific oscillatory phases (Wischnewski, Alekseichuk, and Opitz 2023). Additionally, tACS may induce ephaptic coupling (Anastassiou et al. 2011; Han et al. 2018), a process where non‐synaptic interneural communication occurs via extracellular potential oscillations. The application of tACS thus induces rhythmic changes in the electric field and alternating polarity (excitatory or inhibitory phases). Given that no phosphenes have been reported during monotonous transcranial direct current stimulation (tDCS), it is likely that electrical oscillation plays a crucial role in inducing phosphene perception.

A potential method to test this hypothesis is to differentiate the effects of rhythmic changes in the electric field from those of alternating polarity. To achieve this, the present study employs oscillatory transcranial direct current stimulation (otDCS). In this method, the current oscillation is limited to a single polarity, either positive or negative, thereby removing the influence of polarity switching. Previous studies using otDCS to modulate cognitive function have shown that otDCS can induce changes in excitability (Antal et al. 2008; Bergmann et al. 2009; Groppa et al. 2010) and neural entrainment (Vulić et al. 2021; Živanović et al. 2022), effectively combining features of both tDCS and tACS.

In this study, our first goal was to dissociate the effects of oscillation from polarity. We utilized a range of measurements, including response times, perceived brightness ratings, flash rate, confidence levels, threshold intensity, and pattern drawing indices. This comprehensive approach enabled a thorough investigation of the characteristics of phosphenes induced by both otDCS and tACS, allowing for a direct comparison of potential differences between these stimulation methods. We hypothesized that phosphenes would be observed in both anodal and cathodal otDCS conditions if current oscillation was crucial for inducing phosphene perception.

Our second goal was to investigate the impact of polarity in oscillatory tDCS on phosphene perception. The primary neural effect of tACS was synchronization rather than an overall increase in firing rate (Wischnewski, Alekseichuk, and Opitz 2023). In contrast, the polarity of otDCS might not impact the threshold intensity or interact with the AM frequency. However, polarity might influence ratings on other perceptual measures if it affects the general firing rate. Hsu et al. (2023) explored the characterization of phosphene content by instructing participants to report on the pattern and flash rate of tACS‐induced phosphenes. For flash rate scoring, they observed a linear effect of carrier frequency in sinusoidal tACS. Notably, they found that AM frequency could override the carrier frequency, disrupting the linear relationship and leading to a slower flash rate. These findings suggest that AM may take precedence over carrier frequency in shaping perceptual experiences. Previous studies have shown that the visual neural system can capture envelope information at both the retinal (Ryu et al. 2017) and visual cortical levels (Nguyen et al. 2019; Shapley 1998). Additionally, a simulation study (Negahbani et al. 2018) demonstrated that local field potentials could synchronize with the AM frequency when AM‐tACS was applied to simulated pyramidal cortical cells. Together, these studies suggest that AM information could play a pivotal role in perception by exerting a dominant influence over carrier frequency.

However, an alternative hypothesis could explain the observed slower flash ratings. It is possible that the slower flash perception arises from the waveform of AM‐tACS. Specifically, the amplitude of the carrier oscillation may be modulated by the AM frequency, causing it to vary over time. The amplitude of the carrier oscillation might reach the perception threshold only when the AM frequency peaks at phase angles of 90 or 270°. As a result, AM‐tACS could trigger a phosphene percept at the AM frequency, leading to a slower flash that is less responsive to the carrier frequency. This hypothesis was tested by comparing trial ratings at intensities above the mean threshold. If the phosphene flash followed this pattern, participants were expected to report a higher flash rate during suprathreshold trials, as more oscillations would exceed the phosphene threshold. A null result could indicate that the slower ratings either reflect the perceptual dominance of the AM frequency or data variability and missing values, as not all participants had trials with the same intensity.

Our third goal was to investigate further the source of the slow flash rate in AM‐tACS. To do this, we introduced an additional suprathreshold condition with stimulation at 120% of the threshold intensity. We anticipated that higher stimulation intensity would enhance neural alignment with the oscillation, determining flash perception. If the AM frequency predominated in phosphene perception, the flash rate would align with the AM frequency and remain unaffected by stimulation intensity. Conversely, if the flash rate corresponded to the frequency of carrier oscillations exceeding the perception threshold, ratings for the suprathreshold condition would be faster than those for the threshold condition.

Together, this study investigated the neural mechanisms underlying phosphene perception induced by transcranial electrical stimulation by examining quantitative indicators of phosphene experiences. The specific objectives of this study were to explore (1) the role of neural electric field oscillation by separating the effects of oscillation from polarity, (2) the impact of polarity in oscillatory tDCS on phosphene perception, and (3) the influence of neural alignment with AM frequency on phosphene flash rate through the use of suprathreshold stimulation. By incorporating multiple behavioural measurements beyond the threshold, we aimed to gain a more comprehensive understanding of the neural mechanisms that link neural oscillations with visual perception.

## 2 Method

### 2.1 Participants

Thirty‐seven participants with normal or corrected‐to‐normal vision were recruited for the experiment. Participants with a history of neurological disease or epilepsy or a family history of epilepsy were excluded. Eleven participants did not complete the task because they were unable to detect phosphenes at the maximum intensity (2000 μA) under stimulation conditions. One participant was excluded for consistently responding before the stimulation onset in all conditions. Consequently, data from 25 participants (13 men and 12 women, aged 20 to 45 years) were included in the analysis. All participants were informed about the experimental procedures and provided written consent prior to their participation. All experimental procedures were approved by the Institutional Review Board of Chang‐Gung Memorial Hospital, Taiwan.

### 2.2 Experimental Design and Stimulation Parameters

**FIGURE 1:**
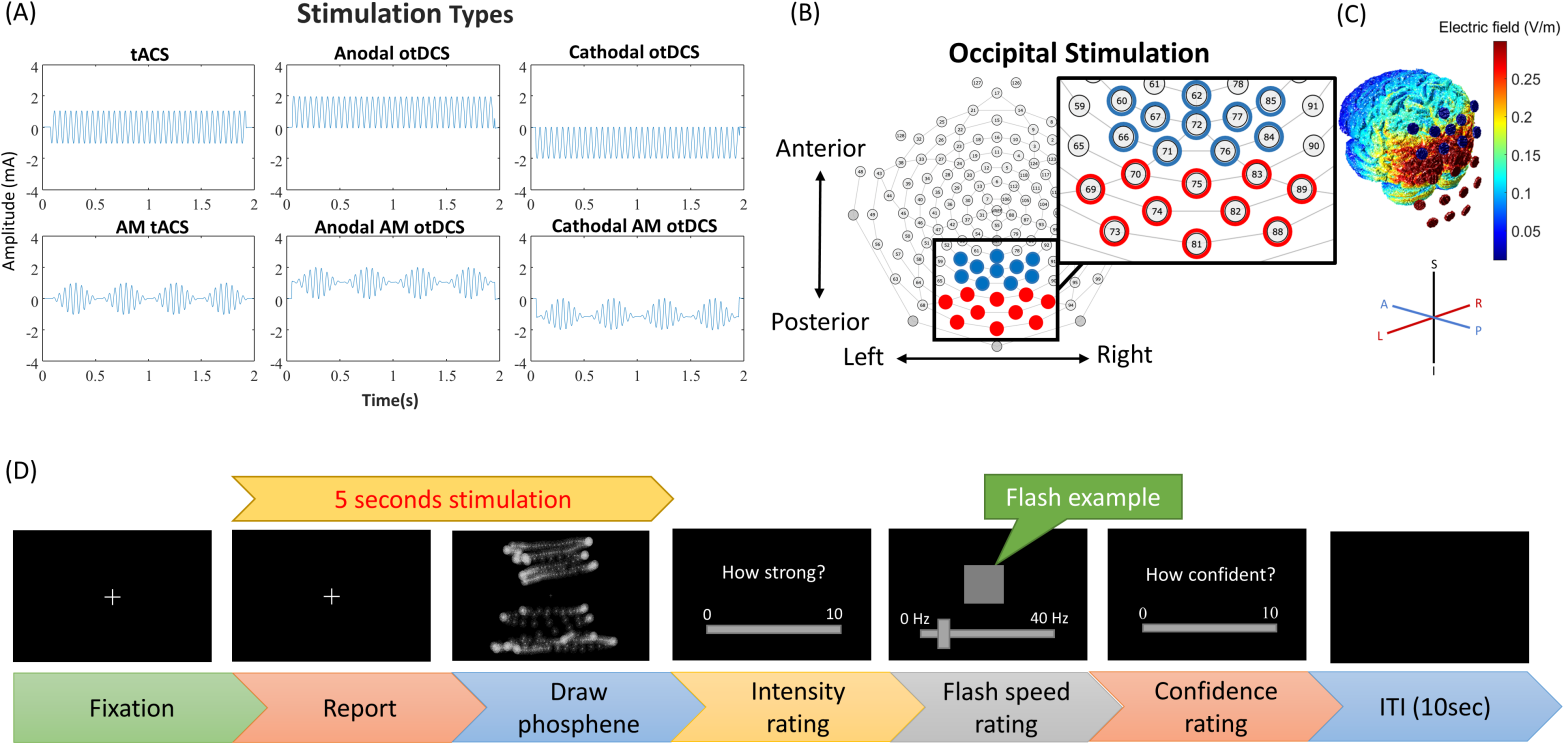
The general diagram of the experimental methods. (A) Illustrations of the given tACS and otDCS waveforms, with the upper row displaying waveforms with the same carrier frequency and the lower row showing waveforms with the same AM frequency. (B) Electrode montages used in the experiment. Electrodes positioned over the occipital scalp were divided into two groups, marked in different colours. In anodal otDCS, the red group served as the anode, and the blue group served as the cathode; this configuration was reversed during cathodal otDCS. In the tACS condition, the two groups of electrodes alternated polarities according to the given waveform. (C) The current distribution simulation was conducted based on the electrode configuration shown in Figure 1B, using an intensity level of 2000 μA. The simulation showed that the occipital stimulation montage produced the highest current density, particularly around the primary visual cortex in the bilateral occipital lobes. The intensity levels of the simulated current are represented by colour coding on the diagrams. The head's current flow was modelled using ROAST software (Huang et al. 2019). (D) Trial structure of the phosphene task. Participants fixated on a central fixation point (0.7° of visual angle) for 2 to 3 s, followed by a 5 s current stimulation. They were instructed to maintain focus on the fixation point and indicate phosphene perception by pressing the space key. Participants then used a mouse to depict the observed phosphene pattern with scattered dots, with the dot positions recorded for subsequent analysis of the pattern's size. Next, participants moved to a new screen to rate the brightness of the flash by adjusting a horizontal slider from 0 (none) to 10 (the strongest). For flash rate scoring, a square at the centre of the screen flashed at frequencies ranging from 0 Hz (left end of the slider) to 40 Hz (right end of the slider). Participants adjusted the slider to match the flash rate of their phosphene to that of the flashing square. Finally, participants rated their confidence level on a scale from 0 (not confident at all) to 10 (very confident).

The current study utilized a within‐subject design, with each participant visiting the lab three times, spaced one week apart. During each visit, participants received stimulation of a single polarity (anodal otDCS, cathodal otDCS, or tACS) across four stimulation blocks: threshold‐level sinusoidal wave (18 Hz), threshold‐level AM wave (2 Hz amplitude‐modulated 18 Hz, 2 AM 18 Hz), suprathreshold sinusoidal wave, and suprathreshold AM wave (waveforms illustrated in Figure 1A). The carrier and AM frequencies were selected based on our previous findings, in which participants reported the highest number of phosphene responses with these frequencies (Hsu et al. 2023). The order of stimulation polarities and blocks was counterbalanced within participants.

### 2.3 Experimental Apparatus

During the experiment, participants were seated in a dimly lit room with a luminance level of 0.25 cd/m^2^, using a chin rest positioned 60 cm from a 24‐in. LCD monitor. The task was programmed using MATLAB 2021b and Psychtoolbox‐3 (Brainard 1997; Kleiner et al. 2007; Pelli 1997), enabling precise control over stimulus presentation. otDCS and tACS were administered using the Geodesic Transcranial Electrical Neuromodulation (GTEN) Planning Module (Magstim Electrical Geodesics, Inc., USA) through a 128‐channel elastic cap. A total of 20 electrodes positioned on the posterior sites were selected for current delivery. The electrode numbers in the EGI system (corresponding to the 10–10 system electrode names) were 60, 66, 67 (PO3), 71, 62 (Pz), 72 (POz), 76, 77 (PO4), 84, 85 (P2), and 69, 73, 70 (O1), 74, 75 (Oz), 81, 82, 83 (O2), 88, 89. Conductive gel was applied to the electrodes to reduce impedance, and an anaesthetic spray was used to minimize tactile sensations induced by stimulation.

For otDCS, the stimulation intensity ranged from 0 to 2000 μA, delivered as a sinusoidal or AM waveform with a positive amplitude for anodal otDCS, and as a waveform between 0 and −2000 μA for cathodal otDCS. For tACS, the stimulus intensity was set to a sinusoidal or AM waveform with an amplitude between −1000 and 1000 μA.

### 2.4 Experimental Procedures

For each condition, the initial tested intensity was set at the maximum output of the equipment, 2000 μA, using the occipital stimulation montage. Participants who could not perceive any phosphene at this intensity were excluded from the experiment. The pre‐experimental stimulation also served to familiarize participants with the experience of phosphenes and the experimental procedures. Threshold intensities were determined using a pre‐experimental test with the modified binary search (MOBS) method (Anderson and Johnson 2006; Tyrrell and Owens 1988). The initial lower and upper bounds were set at 1000 and 2000 μA peak‐to‐peak for anodal otDCS, −1000 and −2000 μA for cathodal otDCS, and −1000 to 1000 μA for tACS.

For each frequency, the first tested intensity was 2000 μA. If a participant reported phosphene perception, the subsequent intensity was set at the midpoint between the highest (2000 μA) and lowest (1000 μA) bounds, i.e., 1500 μA. If phosphene was detected at 1500 μA, the next intensity was the midpoint between the new upper bound (1500 μA) and the previous lower bound (1000 μA). Conversely, if phosphene was not detected at 1500 μA, this value became the new lower bound. The next intensity was set as the midpoint between this new lower bound (1500 μA) and the previous upper bound (2000 μA). This procedure was repeated, adjusting the bounds and testing new midpoint intensities until the participant's response changed from seen to unseen. The intensity at which the phosphene was last detected defined the threshold intensity. This threshold intensity was established through approximately 7–8 trials per condition and subsequently validated through an additional 10 trials to ensure a response rate exceeding 50% in each condition (e.g., Samaha, Gosseries, and Postle 2017). If this criterion was not met, the MOBS procedure was repeated to determine the threshold. Once the threshold was established and confirmed, the suprathreshold intensity was defined as 120% of the validated threshold intensity.

The formal experiment included both threshold and suprathreshold intensities, with 10 trials per condition in each block. In each trial, participants were stimulated for 5 s and asked to press the space key when they perceived a phosphene to record their response time. They then drew the phosphene pattern on the screen using a mouse. After the stimulation, participants entered a new screen to report (1) brightness, (2) flash rate, and (3) confidence level (Figure 1D).

### 2.5 Data Processing and Statistical Methods

In the current study, 2282 response time (RT) trials were collected from 25 participants out of a total of 2890 trials. A total of 94 trials were excluded from further analysis, which included 27 trials with negative RTs (i.e., responses made before stimulation) and 67 trials with RTs either more than 3 standard deviations above or below the mean or exceeding 5 s (i.e., responses made after stimulation). The mean RT for all included trials was 1848.96 ms, with the lowest RT recorded at 204.78 ms and the highest at 4999.33 ms, resulting in an exclusion rate of 4.12%.

For phosphene size estimation, we utilized the “im2bw” and “bwarea” functions in MATLAB 2021b (Pratt 1991). These functions converted phosphene images to grayscale and computed brightness values for pixels ranging from 0 (black) to 1 (white). Pixels with brightness values greater than 0.5 were counted as 1, while others were counted as 0. The phosphene size was calculated as the sum of the counted pixels across the entire screen.

For each measure, we conducted a within‐subject 3 × 2 × 2 ANOVA with the factors of stimulation polarity (anodal otDCS, cathodal otDCS, and tACS), AM condition (0 AM 18 Hz and 2 AM 18 Hz, where 0 Hz AM indicates a sinusoidal waveform), and intensity (threshold: 100% and suprathreshold: 120%). Further paired contrasts were performed when main effects or interactions were significant.

## 3 Results

Repeated measures ANOVAs were conducted on various behavioural parameters, as summarized in Supplementary Table S2.

### 3.1 Threshold Intensity

**FIGURE 2:**
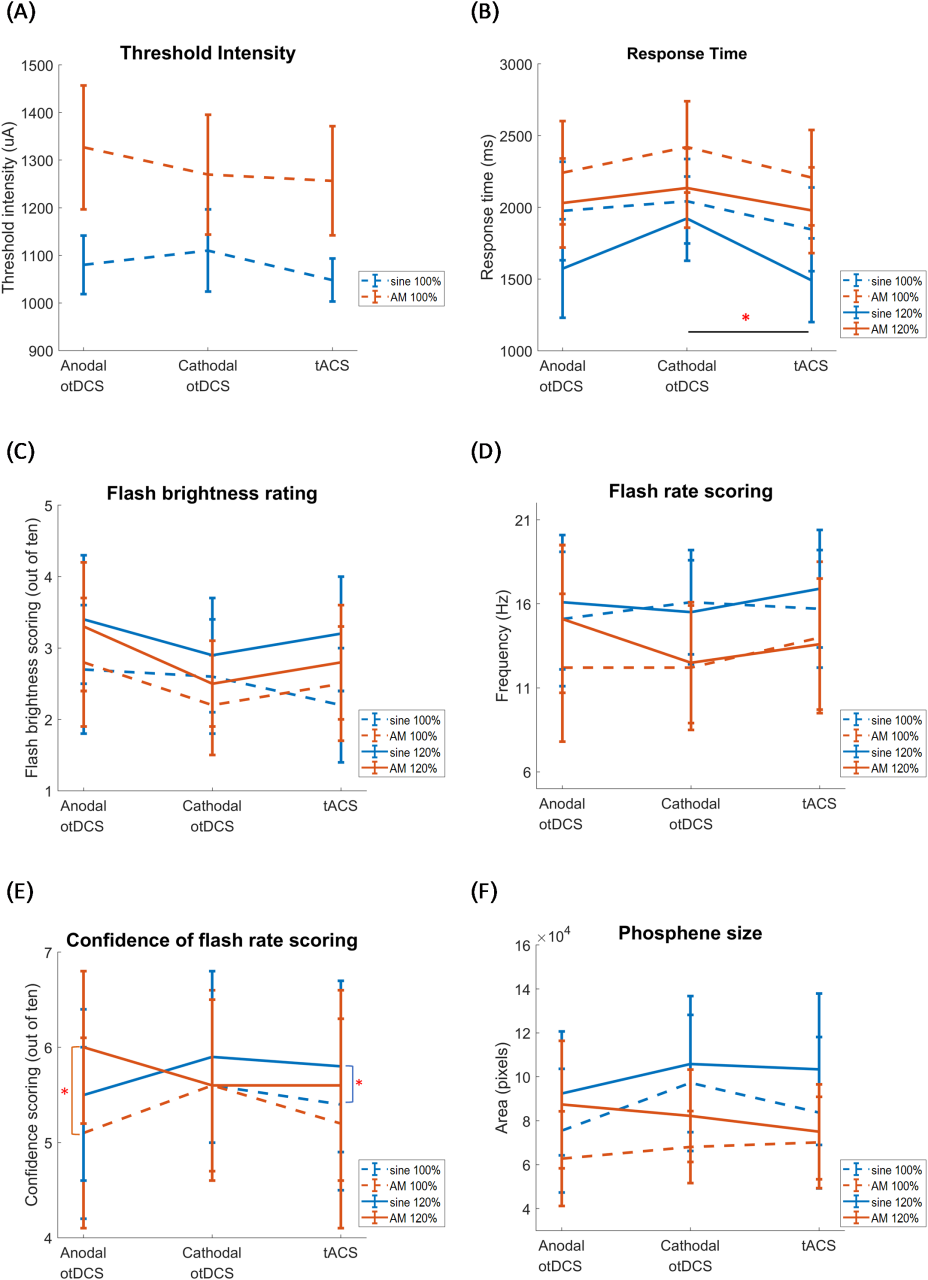
Comparisons of all measurement indices. (A) Phosphene threshold results were calculated by averaging across the anodal otDCS, cathodal otDCS, and tACS conditions, with error bars representing 95% confidence intervals. Phosphene thresholds for AM stimulation were higher than those for sinusoidal stimulation. (B) The graph presents the mean phosphene response time, with the dashed line representing threshold intensity (100%) and the solid line indicating suprathreshold intensity (120%). Phosphene response times for tACS were faster compared to those for cathodal otDCS. Additionally, response times were faster in sinusoidal trials than AM trials and quicker under suprathreshold conditions than under threshold conditions. (C) Flash brightness scoring indicated that participants perceived phosphenes as less intense at threshold than at suprathreshold intensity. Additionally, participants reported lower phosphene brightness under cathodal otDCS and tACS compared to anodal otDCS. (D) The graph displays the mean flash rate scores, showing that AM frequency dominated in both otDCS and tACS conditions. (E) Participants reported higher confidence levels when responding to suprathreshold compared to threshold stimulation. This difference was particularly pronounced in AM stimulation during anodal otDCS and sinusoidal stimulation in tACS. (F) Drawings of phosphene size revealed that sinusoidal stimulation produced significantly larger phosphene areas compared to AM stimulation. Similarly, phosphenes generated at suprathreshold intensities were larger than those at threshold intensities. While both otDCS and tACS did not significantly influence phosphene size, exceptions were observed for AM and intensity levels.

The ANOVA result showed a significant AM main effect (*F* (1,24) = 27.501, *p* < 0.001, *ηp* ^2^ = 0.534). The analysis indicated that the phosphene threshold for AM stimulation (mean value with 95% CI: 1284.33 ± 86.78) was significantly higher than that for sinusoidal stimulation (1079.47 ± 42.62). We did not observe a main effect of polarity (*F* (2,48) = 0.700, *p* = 0.501, *η*_*p*_^2^ = 0.028) or the interactions between polarity and AM (*F* (2,48) = 0.579, *p* = 0.564, *η*_*p*_^2^ = 0.024; Figure 2A).

### 3.2 Response Time

For the reaction time of phosphene response, the ANOVA test revealed a marginally significant main effect of polarity (*F* (2,48) = 2.962, *p =* 0.061, *η*_*p*_^2^ = 0.110). Post‐hoc comparisons with LSD indicated that response time for tACS (1880.96 ± 260.58) was significantly faster than cathodal otDCS (2129.85 ± 234.41, *p* = 0.026), but not anodal otDCS (1954.87 ± 305.11, *p* = 0.489). We found a significant main effect of AM (*F* (1,24) = 27.292, *p <* 0.001, *η*_*p*_^2^ *=* 0.532) and intensity (*F* (1,24) = 43.790, *p* < 0.001, *η*_*p*_^2^ = 0.646), showing that RTs were shorter for the sinusoidal (1808.17 ± 264.18 ms) than the AM (2168.96 ± 230.22 ms) stimulation, and for the suprathreshold (1854.92 ± 241.59 ms) than the threshold (2122.20 ± 240.30 ms) intensity (Figure 2B).

### 3.3 Flash Brightness Rating

ANOVA showed significant main effects of polarity (*F* (1.5,36.1) *=* 4.809, *p =* 0.022, *η*_*p*_^*2*^ *=* 0.167) and intensity (*F* (1,24) = 17.530, *p <* 0.001, *η*_*p*_^*2*^ *=* 0.422), but not AM conditions (*F* (1,24) = 1.798, *p =* 0.192, *η*_*p*_^*2*^ *=* 0.070). The flash brightness ratings were generally higher for the suprathreshold (3.01 ± 0.72) than the threshold (2.50 ± 0.68) intensity. Paired comparison of the three polarities revealed significant results that brightness ratings were higher for anodal otDCS (3.05 ± 0.82) than for cathodal otDCS (2.56 ± 0.64, *p* = 0.025) and tACS (2.65 ± 0.68, *p* = 0.026). The test did not yield any significant two‐way or three‐way interactions (polarity × AM: *F* (2,48) = 1.078, *p =* 0.349, *η*_*p*_^*2*^ *=* 0.043; AM × intensity: *F* (1,24) = 1.131, *p =* 0.298, *η*_*p*_^*2*^ *=* 0.045; polarity × intensity: *F* (2,48) = 1.598, *p =* 0.213, *η*_*p*_^*2*^ *=* 0.062; polarity × AM × intensity: *F* (2,48) = 1.363, *p =* 0.266, *η*_*p*_^*2*^ *=* 0.054) (Figure 2C).

### 3.4 Flash Rate Scoring

The ANOVA revealed a significant main effect of AM (*F* (1,24) = 8.042, *p =* 0.009, *η*_*p*_^*2*^ *=* 0.251) condition, indicating a higher perceived flash rate for the sinusoidal (15.91 ± 3.21) than for the AM (13.26 ± 3.61) stimulations. The main effect of intensity was marginally significant (*F* (1,24) = 3.685, *p =* 0.067, *η*_*p*_^*2*^ *=* 0.133), with a marginal interaction between polarity and intensity (*F* (2,48) *=* 2.697, *p =* 0.078, *η*_*p*_^*2*^ *=* 0.101). Further tests on this effect revealed that only in the anodal otDCS condition did participants score the flash of suprathreshold stimulations as faster than threshold stimulations (*p* = 0.012). However, ANOVA did not reach significance for polarity (*F* (2,48) = 0.380, *p =* 0.686, *η*_*p*_^*2*^ *=* 0.016) or any two‐way and three‐way interactions (polarity × AM: *F* (1.5,35.9) = 0.516, *p* = 0.549, *ηp* ^2^ = 0.021; AM × intensity: *F* (1,24) *=* 0.276, *p =* 0.604, *η*_*p*_^*2*^ *=* 0.011; polarity × AM × intensity: *F* (2,48) *=* 1.320, *p =* 0.277, *η*_*p*_^*2*^ *=* 0.052; Figure 2D).

### 3.5 Confidence of Flash Rate Scoring

ANOVA on the confidence levels revealed a significant main effect of intensity (*F* (1,24) *=* 10.589, *p =* 0.003, *ηp* ^*2*^ *=* 0.306) and the three‐way interaction between polarity, AM, and intensity (*F* (2,48) *=* 3.605, *p =* 0.035, *η*_*p*_^*2*^ *=* 0.131*)*. The statistics yielded a higher confidence rating for the suprathreshold (5.72 ± 0.82) than for the threshold (5.35 ± 0.91) stimulation, and the effect was most pronounced in the AM stimulation of anodal otDCS (*p* = 0.010) and the sinusoidal stimulation of tACS (*p* = 0.034). The analysis did not reveal other main effects or interactions (polarity: *F* (1.5,35.1) *=* 0.691, *p =* 0.464, *η*_*p*_^*2*^ *=* 0.028; AM: *F* (1,24) = 0.134, *p =* 0.717, *η*_*p*_^*2*^ *=* 0.006; polarity × AM: *F* (2,48) *=* 1.725, *p =* 0.189, *η*_*p*_^*2*^ *=* 0.067; polarity × intensity: *F* (2,48) *=* 2.193, *p =* 0.123, *ηp* ^*2*^ *=* 0.084; AM × intensity: *F* (1,24) = 0.015, *p* = 0.904, *η*_*p*_^*2*^ = 0.001; Figure 2E).

### 3.6 Phosphene Size

Statistics on the phosphene area yielded a significant main effect of intensity (*F* (1,24) *=* 12.895, *p =* 0.001, *η*_*p*_^*2*^ *=* 0.350), indicating that the phosphene was perceived larger for the suprathreshold (91,051.20 ± 24,306.02) than the threshold (76,215.75 ± 19,733.47) stimulations. We also found a significant main effect of AM (*F* (1,24) *=* 6.456, *p =* 0.018, *η*_*p*_^*2*^ *=* 0.212*)*, indicating that the phosphene sizes were larger for the sinusoidal (93,014.21 ± 26,324.64) than for AM stimulation (74,252.74 ± 19,156.72). We did not find the main effect of polarity (*F* (1.6,38.1) *=* 0.827, *p =* 0.420, *η*_*p*_^*2*^ *=* 0.033), nor any two‐way (polarity × AM: *F* (2,48) *=* 1.290, *p =* 0.285, *η*_*p*_^*2*^ *=* 0.051; polarity × intensity: *F* (2,48) *=* 0.974, *p =* 0.385, *η*_*p*_^*2*^ *=* 0.039; AM × intensity: *F* (1,24) *=* 0.010, *p =* 0.922, *η*_*p*_^*2*^ *=* 0.000) or three‐way interactions (*F* (1.5,36.3) *=* 1.251, *p =* 0.289, *η*_*p*_^*2*^ *=* 0.050; Figure 2F).

## 4 Discussion

The current study investigates the neural mechanisms underlying phosphene perception induced by otDCS and tACS by manipulating stimulation polarity and intensity and examining their interactions with the AM frequency effect (Hsu et al. 2023). We explored the role of current oscillation in phosphene generation using otDCS and tACS. We hypothesized that if current oscillation were crucial for phosphene perception, then the polarity in otDCS would not interact with the AM condition or stimulation intensity, consistently eliciting phosphene perception.

We also investigated the role of neural alignment in phosphene flash rate by modulating stimulation intensity. A significant interaction between intensity and the AM condition would support the hypothesis that phosphene flash rate perception would be driven solely by alignment to the carrier frequency; otherwise, it would suggest that the AM frequency would be encoded independently of the carrier frequency and would contribute to the perceived flash rate. Finally, the study explored whether the polarity of otDCS affects phosphene perception across different perceptual measures. This exploration may indicate that the underlying mechanisms supporting phosphene perception are influenced by cortical excitability.

Our results, summarized in Table 1, show that the polarity of oscillations did not impact the phosphene threshold, while it influenced response time and brightness ratings. Additionally, polarity interacted with stimulation in flash rate scoring and with both polarity and AM frequency in confidence ratings. AM frequency affected the threshold, response time, and flash rate, but did not influence brightness perception or participants' confidence in their flash rate scoring. Intensity modulated response time, brightness, and confidence levels, but did not affect flash rate scoring. In the following sections, we discuss how these findings address the questions outlined above.

**TABLE 1:** Summary of all effects for each index. The polarity effect indicates whether the current polarity is positive (anodal otDCS), negative (cathodal otDCS), or switching (tACS). AM denotes whether the current is a sinusoidal waveform (18 Hz) or an amplitude‐modulated waveform (2 AM 18 Hz). Intensity specifies whether the stimulation is at threshold (100%) or suprathreshold (120%) amplitude. A mark “X” indicates significant main effects or interactions were found, even if the paired contrasts were not significant for the polarity factor. A mark “0” indicates that no significant effects were observed in the statistical test. ^×^Significant interaction between Polarity, AM, and Intensity.

	Threshold	RT	Brightness	Flash Rate	Confidence
Polarity	0	X	X	0	0
AM	X	X	0	X	0
Intensity	—	X	X	0	X
Interaction	0	0	0	Polarity ^×^	Polarity ^×^
				Intensity	AM ^×^
				(marginal)	Intensity

### 4.1 The Current Oscillation is the Key to Phosphene Occurrence

Consistent with our prediction and findings by Evans, Palmisano, and Croft (2021), participants reported phosphene occurrences in both anodal and cathodal otDCS, with no significant effect of polarity on threshold intensity. This result suggests two key points: (1) current oscillation may be necessary for phosphene generation, and (2) phosphene occurrence is independent of cortical excitability, which is typically modulated by tDCS. In other words, phosphene perception relies on relative changes in the neural electromagnetic field rather than its absolute level.

This finding is plausible, as the phosphene percept primarily involves detecting changes in brightness. By recognizing spatial or temporal changes in brightness, individuals develop perceptions of contours or flashes. This perspective is supported by prosthesis research, which shows that only dynamic stimulation of the visual cortex can elicit meaningful perceptions in individuals who are blind (Beauchamp et al. 2020). The significance of the relative change in phosphene perception aligns with the well‐established predictive coding theory (Rao and Ballard 1999; Schultz, Dayan, and Montague 1997). This theory posits that the brain generates top‐down predictions about what should be perceived. Within this framework, certain neurons convey information about the “difference” between predicted and actual sensory input (Koster‐Hale et al. 2013). Only unexpected inputs are highlighted for further processing (Koch and Poggio 1999), while awareness of information is suppressed if it is “explained away” (Seth, Suzuki, and Critchley 2011).

Our results highlight the importance of neural oscillation in perception generation. Given that tACS applied to human participants at intensities below 2000 μA is generally considered insufficient to induce neural spiking in the visual cortex, the source of phosphenes has remained elusive for the past decade. The prevailing explanation attributes tACS‐induced phosphene perception to the activation of retinal cells through current leakage over the scalp (Laakso and Hirata 2013). However, there is evidence suggesting a contribution from cortical areas, as shown in studies by Evans et al. (2019); Evans, Palmisano, and Croft (2021). Although it is inherently challenging to measure cortical activation related to phosphene perception in vivo in humans, the possibility of cortical involvement cannot be excluded.

Our findings suggest that phosphenes can occur with either type of stimulation, implying that current oscillation may be essential for visual perception, regardless of polarity switching. This phenomenon may be explained by the global resonance theory (McFadden 2021), which proposes that brain electromagnetic field oscillations could serve as the “seat” of consciousness (Hunt 2020; Hunt, Ericson, and Schooler 2022; Hunt and Jones 2023). According to this theory, synchronous neural firing, or neural oscillation, represents a shared electric field where local and global oscillatory couplings may facilitate complex information transfer across different brain areas or levels (Buzsáki 2006). Supporting this perspective, a growing body of research has emphasized the significance of neural oscillations and their functional connectivity in conscious processes (Engel and Fries 2016; Modolo et al. 2020; Plosnić et al. 2023).

### 4.2 Response Time Reflects the Mechanism of Ephaptic Coupling

Response times in our study were measured as the latency between the onset of stimulation and the participants' keypress, indicating they saw a flash. Our findings reveal that response time is influenced by several factors, including stimulation polarity, AM, and intensity. Participants exhibited slower responses during cathodal otDCS compared to anodal otDCS and tACS, in 2 Hz amplitude‐modulated at 18 Hz compared to sinusoidal at 18 Hz, and during threshold compared to suprathreshold stimulations. The effects of both intensity and AM can be attributed to the amount of delivered energy due to waveform shape and amplitude. With the same peak‐to‐peak amplitude, AM stimulation delivers approximately half the energy of sinusoidal stimulation. In our experiment, the threshold amplitude for AM stimulation is about 1.3 times that of sinusoidal stimulation, resulting in delivered energy that is roughly 65% of the threshold for sinusoidal stimulation.

While the relationship between stimulation intensity and response time is commonly observed in psychophysics studies, the underlying neural mechanisms are rarely explained. One possible explanation for the intensity‐RT relationship is ephaptic coupling. Unlike typical synaptic transmission, ephaptic coupling facilitates information transfer between adjacent neurons through extracellular ion exchange or potential oscillation. This mechanism is thought to influence the synchronization of neural spiking (Anastassiou et al. 2011) and may be potent enough to trigger the firing of adjacent pyramidal cells with a single action potential (Han et al. 2018). Computational models suggest that ephaptic coupling within neural bundles may account for selective behaviour (Chawla and Morgera 2014), responses to peripheral nerve stimulation (Capllonch‐Juan and Sepulveda 2020), and the propagation of epilepsy (Shivacharan et al. 2021). A recent simulation study (Schmidt et al. 2021) demonstrated how ephaptic coupling could contribute to response time. The model showed that stronger stimulation induces more neural spiking. While each spike could facilitate ephaptic coupling within the neural bundle, a collection of ephaptic couplings becomes strong enough to elicit spiking volleys, thereby accelerating neural transmission and reducing response time. According to the model, stronger stimuli elicit more neural spiking and prolong the stimulation effect within the neural system.

This ephaptic coupling hypothesis may also explain the slower response time in cathodal otDCS. It is possible that cathodal direct current creates a hyperpolarized environment that hinders the maintenance of spiking volleys. Thus, our response time results may be explained by ephaptic coupling, which is considered a neural mechanism underlying the global resonance theory.

### 4.3 Polarity Effect may Imply the Selective Effect of Stimulation

One of the primary goals of our study was to examine the effect of polarity on phosphene perception. Our results indicated that anodal otDCS induced brighter phosphenes compared to cathodal otDCS and tACS, with no significant difference observed between cathodal otDCS and tACS. However, cathodal otDCS was associated with longer response times. Notably, neither otDCS condition influenced the phosphene threshold, which is typically considered an indicator of neural excitability. This suggests that while the occurrence of phosphenes is driven by electrical oscillation, the perceptual quality can be modulated by cortical excitability.

Transcranial electrical stimulation (TES) has been observed to influence visual perception in various ways. A recent review discussed how non‐invasive current stimulation over the visual cortex can affect visual perception (Bello et al. 2023). Their findings suggest that anodal tDCS might slightly improve contrast perception acutely, and offline current stimulation (including both tACS and anodal tDCS) could significantly enhance contrast detection ability. However, this review did not find any TES effect on response time, which contrasts with our findings.

In a study using the pedestal‐delta‐pedestal psychometric task to explore the contrast detection function of different visual pathways (Costa et al. 2015), a selective effect of TES was found. In the four‐alternative forced‐choice task, participants were asked to detect one of four squares that increased (parvocellular pathway) or decreased (magnocellular pathway) its luminance by a different amount from the other squares for a brief period. The results showed that during anodal tDCS, participants had a higher threshold for detecting targets with decreasing luminance compared to sham stimulation, indicating that anodal tDCS selectively affected the magnocellular pathway. The magnocellular pathway is associated with processing motion, depth, and high temporal and low spatial frequency information. In our study, the impact of polarity on brightness perception might also reflect changes in the magnocellular pathway, as the perception of phosphenes inherently involves detecting temporal contrast in luminance.

### 4.4 Null Effect of Intensity Modulation on Flash Rate Suggests Independence of AM Information Coding in Phosphene Percept

Our flash rate scoring revealed a significant effect in the AM condition, consistent with our previous findings (Hsu et al. 2023). Participants perceived the flash rate as slower with AM stimulation than with sinusoidal stimulation. We observed a marginally significant interaction between intensity and polarity, indicating that the flash rate was perceived as faster under suprathreshold stimulation only in the case of anodal AM otDCS. This effect was not significant in other conditions, suggesting a limited overall impact of intensity on flash rate scoring. Overall, AM emerged as the primary determinant of the perceived flash rate, suggesting that the AM frequency may be encoded by the perceptual system and at least partially contribute to the perceived flash rate of phosphenes. These results suggest that the presence of the AM component plays a critical role in visual perception, particularly in phosphene flash rate.

## 5 Limitations

The current study had three limitations related to trial numbers and experimental design. First, participants underwent stimulation to perceive phosphenes, with thresholds measured using six to seven trials and validated over 10 trials. The suprathreshold intensity was then set at 120% of this threshold. Due to concerns about fatigue and the overall experiment duration, each condition was limited to 10 trials. However, this constraint may have impacted the stability and reliability of the results due to the limited number of trials per condition. Thus, increasing the number of trials in future studies could enhance the robustness of the findings. Second, the experiment utilized a block design, repeating the same stimuli for 10 trials, which raises the concern that participants might make judgements based on prediction rather than direct observation. To address this limitation, future studies could consider using an interleaved trial design and incorporating sham trials to minimize false positives and reduce the likelihood of guessing. Third, a fixation cross was used during the pre‐stimulation phase to reduce eye movement. However, the fixation cross may have had a higher contrast than the phosphene, especially at the threshold level. Consequently, we suggest refraining from employing a fixation cross in future phosphene studies.

## 6 Conclusion

This study investigated the neural mechanisms of phosphene perception using oscillatory transcranial electrical stimulations with different polarities and AM. Our results demonstrated that oscillatory electrical stimulation can effectively elicit phosphenes. Additionally, we showed that neural alignment to AM frequency contributes to the phosphene flash rate, and this effect is independent of the carrier frequency. These findings suggest that alignment to external stimulations significantly influences the subjective perception of temporal frequency. Moreover, the observation of increased brightness with anodal otDCS and prolonged response times with cathodal otDCS indicates a polarity effect on phosphene quality without interacting with threshold intensity. This suggests that current oscillation and polarity may be processed independently, contributing to different aspects of phosphene perception.

In line with our research objectives, the current study provides substantial evidence elucidating the underlying mechanisms of phosphene perception. Our findings in perceptual measurements establish a connection between neural oscillation and subjective visual experiences, thereby opening new possibilities for future research on prosthetic vision techniques.

## Author Contributions

**Che‐Yi Hsu:** conceptualization, data curation, formal analysis, investigation, methodology, resources, software, validation, visualization, writing – original draft. **Tzu‐Ling Liu:** conceptualization, data curation, formal analysis, methodology, resources, validation, visualization, writing – original draft. **Chi‐Hung Juan:** conceptualization, funding acquisition, methodology, project administration.

## Ethics Statement

The study involving human participants was reviewed and approved by the Research Ethics Committee of Chang‐Gung Memorial Hospital, Taiwan. Additionally, all participants provided written informed consent to participate in this study.

## Conflicts of Interest

The authors declare no conflicts of interest.

## Supporting information


**Table S1.** **The summary of mean results.**
**Table S2.** **The summary table of the statistical results.**
**Figure S1.** The results of the response rate. (A). The average response rate results indicated that participants responded above 50% in all conditions and perceived more trials in suprathreshold than in threshold intensity. Additionally, the response rate in the sinusoidal trials was higher than in the AM trials. (B). The bar graph represents each individual data point. The grey dot represents a single participant’s data, and the error bars show the 95% confidence intervals.
**Table S2.** **The summary table of the statistical results.**
**Table S3.** **The statistical results of the reaction time under anodal and cathodal otDCS conditions.**
**Figure S2.** **The results of the response time under anodal and cathodal otDCS conditions.**(A)The results reveal no significant difference between anodal and cathodal otDCS stimulation (*p* = 0.109).
**Table S4.** **The statistical results of the flash rate under anodal and cathodal otDCS conditions.**
**Figure S3.** **The results of the flash rate scoring under anodal and cathodal otDCS conditions.**
**Figure S4.** **Bar graph with each individual data point.**

## Data Availability

The data and analysis code supporting this study's findings are available on the Open Science Framework: https://osf.io/rd4g5/.
