# Supplementary material for: Oscillatory Transcranial Electrical Stimulation and the Amplitude‐Modulated Frequency Dictate the Quantitative Features of Phosphenes
Source: Eur J Neurosci. 2025 Jan 8;61(1):e16658. doi: 10.1111/ejn.16658 (PMC11708813; doi:10.1111/ejn.16658)
Supplement: Supplementary file 1 — Table S1. The summary of mean results. Table S2. The summary table of the statistical results. Figure S1. The results of the response rate. (A). The average response rate results indicated that participants responded above 50% in all conditions and perceived more trials in suprathreshold than in threshold intensity. Additionally, the response rate in the sinusoidal trials was higher than in the AM trials. (B). The bar graph represents each individual data point. The grey dot represents a single participant’s data, and the error bars show the 95% confidence intervals. Table S2. The summary table of the statistical results. Table S3. The statistical results of the reaction time under anodal and cathodal otDCS conditions. Figure S2. The results of the response time under anodal and cathodal otDCS conditions.(A)The results reveal no significant difference between anodal and cathodal otDCS stimulation (p = 0.109). Table S4. The statistical results of the flash rate under anodal and cathodal otDCS conditions. Figure S3. The results of the flash rate scoring under anodal and cathodal otDCS conditions. Figure S4. Bar graph with each individual data point. [file EJN-61-0-s001.pdf]

Supplementary Materials

Table S1. The summary of mean results

	Intensity (%)	Anodal otdcs (mean(std.))		Cathodal otdcs (mean(std.))		tACS (mean(std.))	
		18 Hz	2AM18Hz	18 Hz	2AM18Hz	18 Hz	2AM18Hz
Threshold intensity (uA) Mean (min~max)	100	1080.1(148.4) (1003~1527)	1326.8(315.2) (1003~1922)	1110.2 (208.5) (1003~1699)	1269.6(305.1) (1003~1875)	1048.2(109.7) (1006~1506)	1256.6(277.3) (1006~2000)
Response rate (out of 10)	100	7.3 (2.9) (3~10)	6.8 (3.1) (2~10)	7.3 (2.4) (2~10)	6.7 (2.4) (3~10)	7.2 (3.2) (1~10)	6.9 (2.8) (2~10)
	120	8.4 (2.4) (3~10)	7.7 (2.4) (2~10)	8.5 (1.9) (4~10)	7.8 (2.2) (3~10)	8.6 (2.3) (2~10)	8.0 (2.2) (2~10)
Reaction time (ms)	100	1974.9 (884.3) (620.0~ 3372.0)	2241.6 (872.1) (1048.4~ 3834.0)	2042.1 (748.8) (802.2~3790.1)	2421.1 (771.0) (1005.6~3991.0)	1846.2 (769.8) (767.5~ 4005.8)	2207.2 (806.1) (1085.3~ 3819.7)
	120	1573.3 (832.6) (496.0~3593.0)	2029.7 (752.4) (884.6~3761.4)	1921.3 (713.2) (768.3~3778.1)	2134.8 (668.6) (697.6~ 3774.0)	1491.1 (706.9) (663.8~3355.2)	1979.3 (724.5) (994.1~ 3480.9)
Flash brightness rating (out of 10)	100	2.7 (1.9) (0.1~8.0)	2.8 (2.2) (0.0~10)	2.6 (1.9) (0.0~7.3)	2.2 (1.7) (1.0~8.4)	2.2 (1.4) (0.3~5.0)	2.5 (1.8) (0.3~7.8)
	120	3.4 (2.1) (1.0~9.5)	3.3 (2.2) (0.3~9.0)	2.9 (2.0) (0.6~8.6)	2.5 (1.5) (0.9~5.3)	3.2 (2.0) (1.0~9.1)	2.8 (2.0) (1.0~9.1)
Flash rate scoring (Hz)	100	15.1 (10.4) (1.8~38.5)	12.2 (10.7) (0.5~38.8)	16.1 (7.8) (3.8~33.1)	12.2 (8.9) (1.0~29.9)	15.7 (9.5) (2.2~39.0)	14.0 (11.0) (1.3~37.5)
	120	16.1 (9.7) (4.5~37.8)	15.1 (10.6) (0.7~37.0)	15.5 (7.5) (1.2~33.9)	12.5 (8.6) (1.4~30.9)	16.9 (8.6) (3.7~35.9)	13.6 (9.5) (2.2~37.6)
Confidence of flash rate scoring (out of 10)	100	5.1 (2.4) (1.0~9.5)	5.1 (2.4) (1.0~10.0)	5.6 (2.3) (1.3~10.0)	5.6 (2.4) (0.7~10.0)	5.4 (2.2) (1.0~10.0)	5.2 (2.6) (1.0~10.0)
	120	5.5 (2.3) (1.0~9.5)	6.0 (1.8) (3.0~9.0)	5.9 (2.2) (1.0~10.0)	5.6 (2.2) (0.3~10.0)	5.8 (2.3) (1.0~10.0)	5.6 (2.5) (0.8~10.0)
Phosphene size (pixels)	100	7.6 (5.2)×10^4 (1.3~20.1)×10^4	6.3 (5.2)×10^4 (1.5~27.9)×10^4	9.7 (8.0)×10^4 (3.1~31.9)×10^4	6.8 (4.0)×10^4 (1.6~17.1)×10^4	8.4 (8.0)×10^4 (1.6~34.4)×10^4	7.0 (5.0)×10^4 (1.0~20.9)×10^4
	120	9.2 (6.8)×10^4 (2.0~29.5)×10^4	8.7 (7.0)×10^4 (2.4~37.4)×10^4	10.6 (7.5) (3.8~29.4)×10^4	8.2 (5.1)×10^4 (2.2~21.3)×10^4	10.3 (8.4)×10^4 (2.6~35.8)×10^4	7.5 (5.2)×10^4 (2.8~28.9)×10^4

Table S2. The summary table of the statistical results

Response rate	SP	$F(2,48) = 0.161$, $p = 0.851$, $\eta_p^2 = 0.007$
	AM	$F(1,24) = 11.558$, $p = 0.002$, $\eta p^2 = 0.325$ **
	IT	$F(1,24) = 33.060$, $p < 0.001$, $\eta_p^2 = 0.579$ ***
	SP × AM	$F(1.4,33.4) = 0.039$, $p = 0.912$, $\eta_p^2 = 0.002$
	SP × IT	$F(2,48) = 0.249$, $p = 0.781$, $\eta_p^2 = 0.010$
	AM × IT	$F(1,24) = 0.437$, $p = 0.515$, $\eta_p^2 = 0.018$
	SP × AM × IT	$F(2,48) = 0.085$, $p = 0.918$, $\eta_p^2 = 0.004$

Note. Stimulation polarity (SP), AM conditions (AM), and Intensity (IT). *: $p < 0.05$, **: $p < 0.01$. ***: $p < 0.001$. η_p^2 : partial eta squared.

The three-way ANOVA revealed a significant main effect of AM ($F(1,24) = 11.558$, $p = 0.002$, $\eta p^2 = 0.325$), and intensity ($F(1,24) = 33.060$, $p < 0.001$, $\eta_p^2 = 0.579$), but not stimulation polarity conditions ($F(2,48) = 0.161$, $p = 0.851$, $\eta_p^2 = 0.007$), indicating a higher response rate for sinusoidal (mean value with 95% CI: 7.88 ± 0.80) than for AM (7.28 ± 0.85), and for supra-threshold (8.14 ± 0.72) than the threshold (7.02 ± 0.93) intensity. However, ANOVA did not reveal the significance of any two-way and three-way interactions (polarity × AM: $F(1.4,33.4) = 0.039$, $p = 0.912$, $\eta_p^2 = 0.002$; AM × intensity: $F(1,24) = 0.437$, $p = 0.515$, $\eta_p^2 = 0.018$; polarity × AM × intensity: $F(2,48) = 0.085$, $p = 0.918$, $\eta_p^2 = 0.004$).

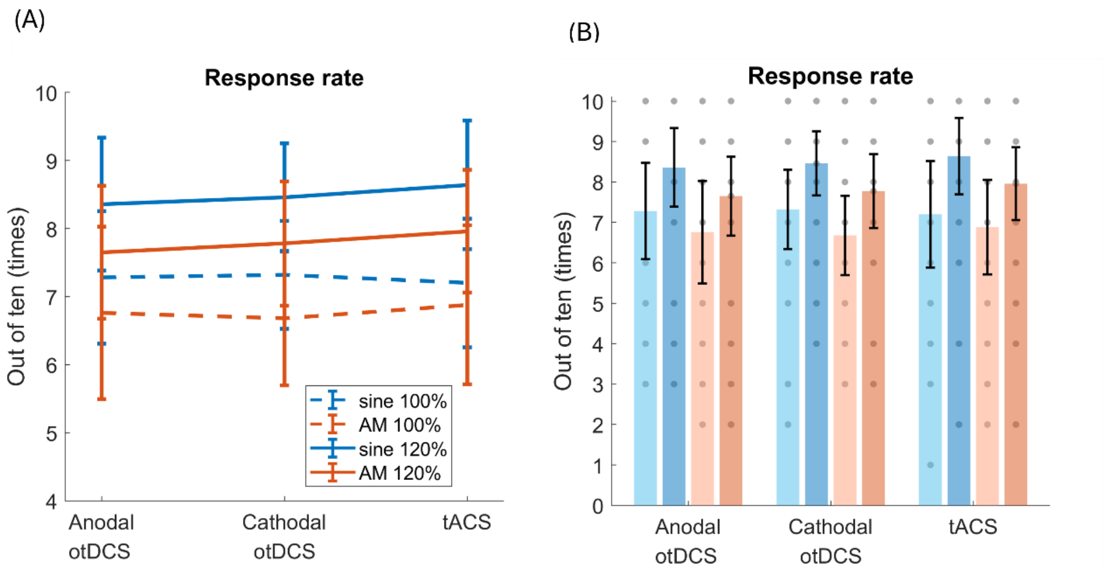

Figure S1. The results of the response rate. (A). The average response rate results indicated that participants responded above 50 percent in all conditions and perceived more trials in suprathreshold than in threshold intensity. Additionally, the response rate in the sinusoidal trials was higher than in the AM trials. (B). The bar graph represents each individual data point. The gray dot represents a single participant's data, and the error bars show the 95% confidence intervals.

Table S2. The summary table of the statistical results

Threshold intensity	SP	$F(2,48) = 0.700, p = 0.501, \eta_p^2 = 0.028$
	AM	$F(1,24) = 27.501, p < 0.001, \eta_p^2 = 0.534$ ***
	SP × AM	$F(2,48) = 0.579, p = 0.564, \eta_p^2 = 0.024$
Reaction time	SP	$F(2,48) = 2.962, p = 0.061, \eta_p^2 = 0.110$
	AM	$F(1,24) = 27.292, p < 0.001, \eta_p^2 = 0.532$***
	IT	$F(1,24) = 43.790, p < 0.001, \eta_p^2 = 0.646$ ***
	SP × AM	$F(2,48) = 0.312, p = 0.733, \eta_p^2 = 0.013$
	SP × IT	$F(2,48) = 0.348, p = 0.708, \eta_p^2 = 0.014$
	AM × IT	$F(1,24) = 0.342, p = 0.564, \eta_p^2 = 0.014$
	SP × AM × IT	$F(1.5,37.0) = 1.169, p = 0.311, \eta_p^2 = 0.046$
Response rate	SP	$F(2,48) = 0.161, p = 0.851, \eta_p^2 = 0.007$
	AM	$F(1,24) = 11.558, p = 0.002, \eta p^2 = 0.325$ **
	IT	$F(1,24) = 33.060, p < 0.001, \eta_p^2 = 0.579$ ***
	SP × AM	$F(1.4,33.4) = 0.039, p = 0.912, \eta_p^2 = 0.002$
	SP × IT	$F(2,48) = 0.249, p = 0.781, \eta_p^2 = 0.010$
	AM × IT	$F(1,24) = 0.437, p = 0.515, \eta_p^2 = 0.018$
	SP × AM × IT	$F(2,48) = 0.085, p = 0.918, \eta_p^2 = 0.004$
Flash brightness rating	SP	$F(1.5,36.1) = 4.809, p = 0.022, \eta_p^2 = 0.167$ *
	AM	$F(1,24) = 1.798, p = 0.192, \eta_p^2 = 0.070$
	IT	$F(1,24) = 17.530, p < 0.001, \eta_p^2 =0.422$ ***
	SP × AM	$F(2,48) = 1.078, p = 0.349, \eta_p^2 = 0.043$
	SP × IT	$F(2,48) = 1.598, p = 0.213, \eta_p^2 = 0.062$
	AM × IT	$F(1,24) = 1.131, p = 0.298, \eta_p^2 = 0.045$
	SP × AM × IT	$F(2,48) = 1.363, p = 0.266, \eta_p^2 = 0.054$
Flash rate scoring	SP	$F(2,48) = 0.380, p = 0.686, \eta_p^2 = 0.016$
	AM	$F(1,24) = 8.042, p = 0.009, \eta_p^2 = 0.251$ **
	IT	$F(1,24) = 3.685, p = 0.067, \eta_p^2 = 0.133$
	SP × AM	$F(1.5,35.9) = 0.516, p = 0.549, \eta p^2 = 0.021$
	SP × IT	$F(2,48) = 2.697, p = 0.078, \eta_p^2 = 0.101$

	AM × IT	$F(1,24) = 0.276$, $p = 0.604$, $\eta_p^2 = 0.011$
	SP × AM × IT	$F(2,48) = 1.320$, $p = 0.277$, $\eta_p^2 = 0.052$
Confidence of flash rate scoring	SP	$F(1.5,35.1) = 0.691$, $p = 0.464$, $\eta_p^2 = 0.028$
	AM	$F(1,24) = 0.134$, $p = 0.717$, $\eta_p^2 = 0.006$
	IT	$F(1,24) = 10.589$, $p = 0.003$, $\eta_p^2 = 0.306$ **
	SP × AM	$F(2,48) = 1.725$, $p = 0.189$, $\eta_p^2 = 0.067$
	SP × IT	$F(2,48) = 2.193$, $p = 0.123$, $\eta_p^2 = 0.084$
	AM × IT	$F(1,24) = 0.015$, $p = 0.904$, $\eta_p^2 = 0.001$
	SP × AM × IT	$F(2,48) = 3.605$, $p = 0.035$, $\eta_p^2 = 0.131$ *
Phosphene size	SP	$F(1.6,38.1) = 0.827$, $p = 0.420$, $\eta_p^2 = 0.033$
	AM	$F(1,24) = 6.456$, $p = 0.018$, $\eta_p^2 = 0.212$ *
	IT	$F(1,24) = 12.895$, $p = 0.001$, $\eta_p^2 = 0.350$ **
	SP × AM	$F(2,48) = 1.290$, $p = 0.285$, $\eta_p^2 = 0.051$
	SP × IT	$F(2,48) = 0.974$, $p = 0.385$, $\eta_p^2 = 0.039$
	AM × IT	$F(1,24) = 0.010$, $p = 0.922$, $\eta_p^2 = 0.000$
	SP × AM × IT	$F(1.5,36.3) = 1.251$, $p = 0.289$, $\eta_p^2 = 0.050$

Note. Stimulation polarity (SP), AM conditions (AM), and Intensity (IT). *: $p < 0.05$, **: $p < 0.01$. ***: $p < 0.001$. η_p^2 : partial eta squared.

Table S3. The statistical results of the reaction time under anodal and cathodal otDCS conditions

Reaction time	SP	$F(1,24) = 2.770$, $p = 0.109$, $\eta_p^2 = 0.103$
	AM	$F(1,24) = 11.078$, $p = 0.003$, $\eta_p^2 = 0.316$ **
	IT	$F(1,24) = 19.946$, $p < 0.001$, $\eta_p^2 = 0.454$ ***
	SP × AM	$F(1,24) = 0.198$, $p = 0.660$, $\eta_p^2 = 0.008$
	SP × IT	$F(1,24) = 0.673$, $p = 0.420$, $\eta_p^2 = 0.027$
	AM × IT	$F(1,24) = 0.010$, $p = 0.921$, $\eta_p^2 = 0.000$
	SP × AM × IT	$F(1,24) = 2.921$, $p = 0.100$, $\eta_p^2 = 0.108$

Note. Stimulation polarity (SP: anodal otDCS and cathodal otDCS), AM conditions (AM), and Intensity (IT). *: $p < 0.05$, **: $p < 0.01$. ***: $p < 0.001$. η_p^2 : partial eta squared.

1. For the reaction time of phosphene response, the three-way ANOVA test on the otDCS levels reveal a significant main effect of AM ($F(1,24) = 11.078$, $p = 0.003$, $\eta_p^2 = 0.316$, mean value with 95% CI: Sinusoidal: 1877.93 ± 293.44 ms; Amplitude modulation: 2206.80 ± 243.27 ms), intensity ($F(1,24) = 19.946$, $p < 0.001$, $\eta_p^2 = 0.454$, threshold intensity: 2169.94 ± 259.41 ms; suprathreshold intensity: 1914.79 ± 253.29 ms), but not polarity ($F(1,24) = 2.770$, $p = 0.109$, $\eta_p^2 = 0.103$, anodal otDCS: 1954.87 ± 305.11 ms; cathodal otDCS: 2129.85 ± 234.41 ms). We did not find a significant three-way interaction between polarity, AM, and intensity ($F(1,24) = 2.921$, $p = 0.100$, $\eta_p^2 = 0.108$).

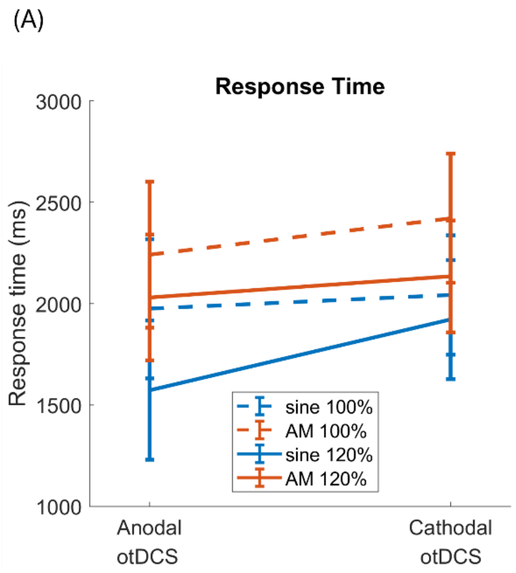

Figure S2. The results of the response time under anodal and cathodal otDCS conditions. (A) The results reveal no significant difference between anodal and cathodal otDCS stimulation ($p = 0.109$).

Table S4. The statistical results of the flash rate under anodal and cathodal otDCS conditions

Flash rate scoring	SP	$F(1,24) = 0.220$, $p = 0.643$, $\eta_p^2 = 0.009$
	AM	$F(1,24) = 5.669$, $p = 0.026$, $\eta_p^2 = 0.191$ *
	IT	$F(1,24) = 5.205$, $p = 0.032$, $\eta_p^2 = 0.178$ *
	SP × AM	$F(1,24) = 0.701$, $p = 0.411$, $\eta_p^2 = 0.028$
	SP × IT	$F(1,24) = 4.004$, $p = 0.057$, $\eta_p^2 = 0.143$
	AM × IT	$F(1,24) = 1.587$, $p = 0.220$, $\eta_p^2 = 0.062$
	SP × AM × IT	$F(1,24) = 0.263$, $p = 0.613$, $\eta_p^2 = 0.011$

Note. Stimulation polarity (SP: anodal otDCS and cathodal otDCS), AM conditions (AM), and Intensity (IT). *: $p < 0.05$, **: $p < 0.01$. ***: $p < 0.001$. ηp^2: partial eta squared.

3. The three-way ANOVA test on the otDCS levels revealed a significant main effect of AM ($F(1,24) = 5.669$, $p = 0.026$, $\eta p^2 = 0.191$), intensity ($F(1,24) = 5.205$, $p = 0.032$, $\eta p^2 = 0.178$), but not polarity differences ($F(1,24) = 0.220$, $p = 0.643$, $\eta_p^2 = 0.009$). We also found a marginal interaction between polarity and intensity ($F(1,24) = 4.004$, $p = 0.057$, $\eta_p^2 = 0.143$). Further tests on this effect revealed that only in the anodal otDCS participants score the flash of suprathreshold stimulations faster than threshold stimulations ($p = 0.012$). However, ANOVA did not reveal the significance of any 2-way and 3-way interactions (polarity × AM: $F(1,24) = 0.701$, $p = 0.411$, $\eta_p^2 = 0.028$; AM × intensity: $F(1,24) = 1.587$, $p = 0.220$, $\eta_p^2 = 0.062$; polarity × AM × intensity: $F(1,24) = 0.263$, $p = 0.613$, $\eta_p^2 = 0.011$).

(A)

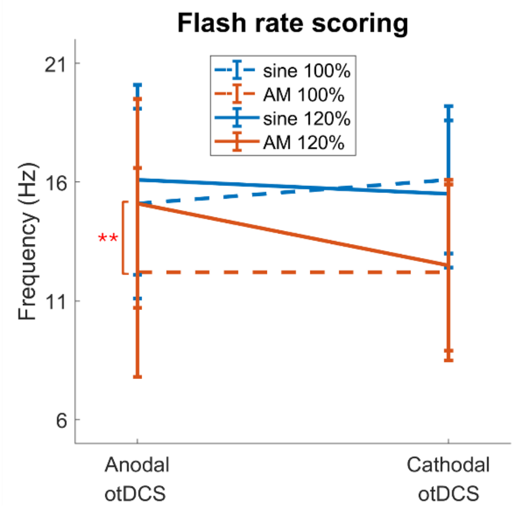

Figure S3. The results of the flash rate scoring under anodal and cathodal otDCS conditions. (A) Anodal AM otDCS resulted in the flash rate effect at suprathreshold intensity being inconsistent with that at threshold intensity. The results revealed that the flash frequency was faster under suprathreshold intensity than at threshold in the anodal AM otDCS condition ($p = 0.012$).

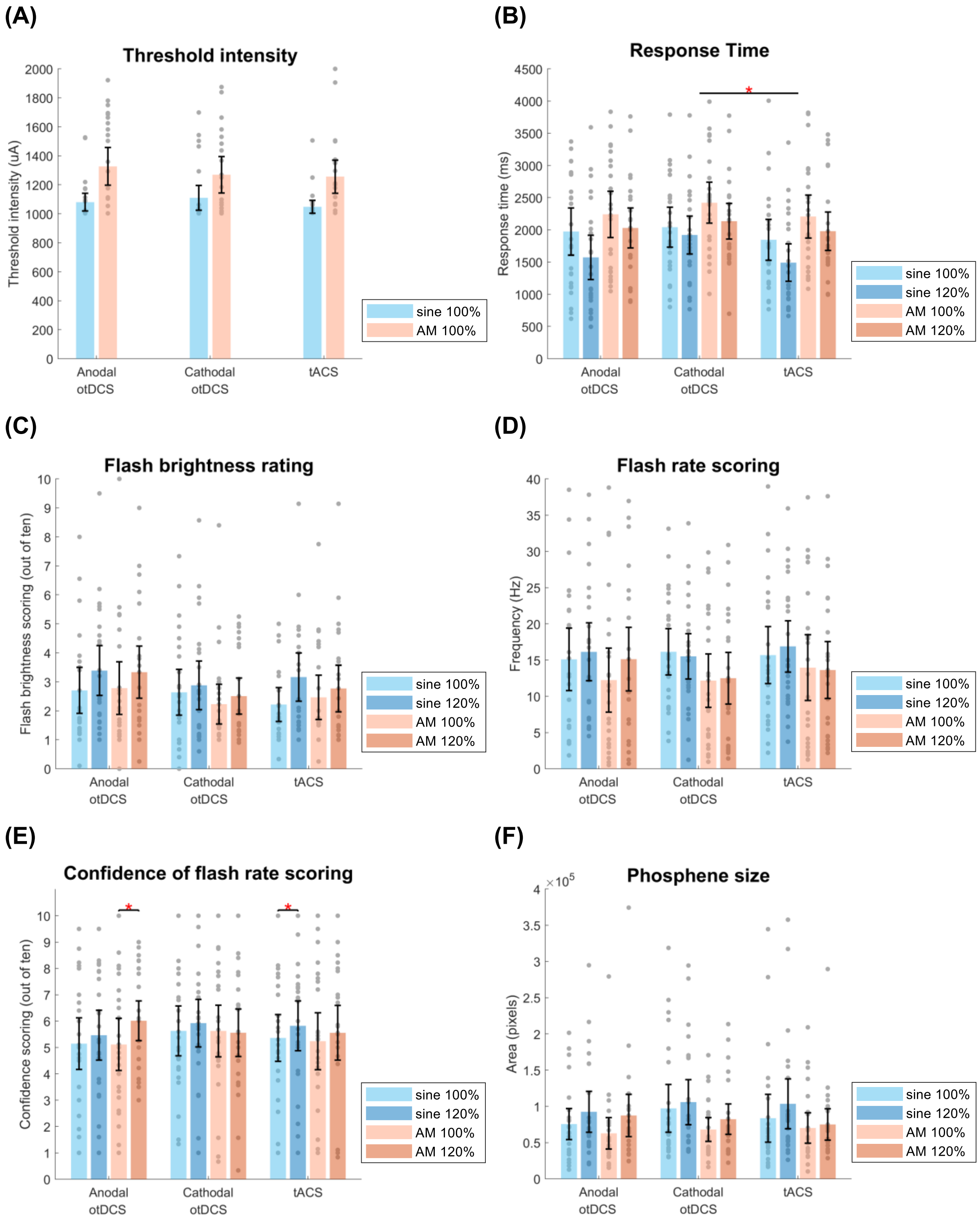

Figure S4. Bar graph with each individual data point. The gray dot represents a single participant's data. The error bars represent the 95% confidence intervals, and the red asterisk indicates a significant difference ($p < 0.05$).
